# Using social network analysis to plan, promote and monitor intersectoral collaboration for health in rural India

**DOI:** 10.1371/journal.pone.0219786

**Published:** 2019-07-17

**Authors:** Connie Hoe, Binita Adhikari, Douglas Glandon, Arindam Das, Navpreet Kaur, Shivam Gupta

**Affiliations:** 1 Department of International Health, Johns Hopkins Bloomberg School of Public Health, Baltimore, Maryland, United States of America; 2 Symbiosis Institute of Health Sciences, Pune, India; 3 HCL Foundation, Uttar Pradesh, India; University of North Carolina at Greensboro Center for Women’s Health and Wellness, UNITED STATES

## Abstract

**Background:**

As population health and well-being are influenced by multiple factors that cut across sectoral boundaries, an intersectoral approach that acknowledges and leverages the multiple determinants, actors and sectors at play is increasingly seen as critical for achieving meaningful and lasting improvements. In this study, we utilize social network analysis (SNA) to characterize the intersectoral collaboration between the organizations working on maternal & child health (MCH) and water & sanitation (WASH) before and immediately after the implementation of HCL Foundation (HCLF)-funded *HCL Samuday Project* (2015–2017) in a rural block of Uttar Pradesh state, India. While SNA has been used to examine public health issues, few have used it monitor stakeholder relationships, intervene, improve and facilitate project implementation involving intersectoral partnerships, particularly in the context of a low-and middle-income countries.

**Method:**

An organization-level SNA was conducted with 31 key informants from 24 organizations working on MCH and/or WASH in Kachhauna, Uttar Pradesh, India. Data were collected using face-to-face, semi-structured interviews between June and September 2017. Density, centrality and homophily were calculated to describe the network and a qualitative analysis was also conducted to identify the strengths and weaknesses of collaboration between organizations working on MCH and WASH.

**Results:**

Overall, our findings showed that the network of organizations working on MCH and WASH in Kachhauna grew in number since the implementation of *Samuday*. HCLF rapidly achieved centrality, thus positioning the organization to serve as a gatekeeper of information and enabling it to play a coordinator role within the network. Direct collaboration between other organizations working on MCH and WASH was low at both time points. Interviews with key informants indicated widespread interest in increasing interorganizational interactions and engagement throughout the network.

**Conclusion:**

This study demonstrates the feasibility and practical application of SNA for projects like *Samuday* that involve intersectoral collaboration. It also provides lessons about the use of SNA with organizations as the unit of analysis and in the context of rural India, including challenges, practical considerations, and limitations.

## Introduction

Population health and well-being are influenced by multiple factors that cut across sectoral boundaries, including political, economic, social and environmental determinants [1–4]. As these complex set of factors frequently lie outside of the scope of the health sector, an intersectoral approach that acknowledges and leverages the multiple determinants, actors and sectors at play is increasingly seen as critical for achieving meaningful and lasting improvements [1]. The World Health Organization (WHO) has promoted such an approach and the 1978 Alma-Ata Declaration on Primary Health Care, clearly emphasized that:

“All governments should formulate national policies, strategies and plans of action to launch, and sustain primary health care as part of a comprehensive national health system and in coordination with other sectors.*”*

*(Article VIII)* [5]

More recently, in 2008, the Commission on the Social Determinants of Health issued a report underscoring the importance of addressing social and economic determinants in order to improve health equity, recommending that ministries of health engage with non-health sectors [1]. Similarly, in 2009, the World Health Assembly Resolution, *Reducing Health Inequities through Action on the Social Determinants of Health*, urged governments to adopt a *Health in All Policies* approach to better address the complex set of factors that can affect health and equity [6].

While an intersectoral approach is vital for all countries, it is of particular importance for low-and middle-income countries (LMICs) where the health sector predominantly focuses on the provision of health care services, and collaboration across sectors remains underdeveloped [7].

In this study, we focus on India, a lower middle-income country, that has strategically pursued an intersectoral approach to tackle health inequities, particularly between urban and rural areas. Various programs have been launched by the government to improve service delivery of health, nutrition, water, and sanitation by mandating collaboration across different sectors. For example, the two initiatives—the Integrated Child Development Services (ICDS) under the Ministry of Women and Children Development and the National Rural Health Mission (NRHM) under the Ministry of Health and Family Welfare—helped improve convergence between the two ministries as well as service delivery through increased community participation [8]. Similarly, Prasad et al (2013) revealed that rural infant mortality rates in India fell by 15.6 percentage points between 2004 and 2011 in high-focus states of NRHM, further demonstrating the importance of intersectoral action in such contexts [9].

### HCL Samuday Project

In 2015, HCL Foundation (HCLF) launched *HCL Samuday Project* to enhance rural health and well-being in India. This rural development project targets multiple sectors (e.g. maternal & child health (MCH) as well as water & sanitation (WASH)), beyond the scope of ICDS and NRHM, and establishes partnerships with central and state governments, village communities, non-governmental organizations, as well as other stakeholders to build model villages across Uttar Pradesh (UP), India. UP was selected as it is the most populous state of the country with an estimated population of about 224 million in 2017; it also has some of the worst health and well-being indicators [10].

*HCL Samuday Project* is being implemented in a phased approach over multiple administrative blocks of UP, starting with Kachauuna block in the Hardoi district, one of the high priority districts identified by the Government of India for efforts to rapidly improve health outcomes [11]. Only 13.5% of women in the Kachhauna block, for example, received over four antenatal check visits during pregnancy [12] compared to 21.7% at the state level [10], and 44.8% at the national level. Likewise, only 35% of the households have access to toilet in Kachhauna [12] while this is similar to the prevalence at the state level, it is less than the national level of 48.4% [13].

The primary aims of this study are to 1) utilize social network analysis (SNA) to characterize the intersectoral collaboration between the organizations working on MCH and WASH before and immediately after the implementation of *HCL Samuday Project* (2015–2017), and 2) provide lessons about the use of SNA with organizations as the unit of analysis and in the context of rural India, including challenges, practical considerations, and limitations.

SNA is a methodological approach that measures and maps relationships among social units (e.g. individuals, groups, organizations). With roots in classical sociology and anthropology, SNA assumes that actors are embedded in social systems and interconnected as a result. One of the key elements in a SNA is a graphical depiction of links between these social units called the *sociogram*. These *sociograms* allow for the visual examination of the size, structure and attributes of the network and can also yield several multiple quantitative measures that can be monitored over time [14–16]. To date, SNA has been used to examine an array of public health issues including obesity and tobacco use in both high-income and LMICs [17–18]. These studies, however, have predominantly used individuals as the unit of analysis. While experts have urged for the use of SNA in monitoring stakeholder relationships, intervening, improving and facilitating project implementation involving intersectoral partnerships [19], few studies have employed this tool to this effect. To our knowledge, of the few studies that currently exist, most were conducted in high-income countries [20–21].

SNA can add substantial value to projects like *HCL Samuday* as results can prompt project implementers to understand which actors are doing what, think more carefully about how they can and should engage with other partners, provide a structured framework for conceptualizing and planning collaboration, and monitor the status of those relationships, comparing them to what was planned, and identifying the changes required.

## Methods

This cross-sectional study was conducted between June and September 2017, using face-to-face, semi-structured interviews. As a comprehensive list of organizations working on MCH and/or WASH in Kachhauna did not exist, *snowball sampling* [22] was employed to identify organizations working on MCH and/or WASH in Kachhauna. We define *organization* as groups of people who are working together to pursue a collective goal [23]. Large organizations, such as government ministries, that have different levels (e.g. district, block, etc.) were treated as separate organizations. Accordingly, individuals or organizations that only supplied products for the project were excluded.

The study team requested HCLF staff to identify partner organizations with whom they work on MCH and WASH interventions. They were also asked to list organizations whom they do not work with in the two sectors. The term “work” was defined loosely, ranging from informal contact (through email, phone call, or in person meetings) to formal work agreements. Subsequently, for each organization, a representative who is knowledgeable about the organization’s work was identified as the key informant for the interview. For some organizations, particularly the ones that worked on both MCH and WASH, more than one representative was interviewed; in these circumstances responses were based on group consensus. During these interviews, individuals were asked to also identify other organizations they work with. On average interviews lasted around 30 minutes and were conducted in English and/or Hindi as per the preference of the informant.

### Data analysis

For the quantitative social network analysis component, network data were first entered into a Microsoft Excel spreadsheet whereby the names of organizations were recorded in the rows and the presence or absence of a working relationship based on the aforementioned interviews was documented in the columns. A second matrix was developed to include the attributes of the organizations, including the sector(s) in which they worked (MCH and/or WASH) and type of organization (e.g. public, private, international). Subsequently, sociograms were created using NetDraw [24] to enable visual examination of the size, structure and attributes of the network before and after the implementation of *HCL Samuday Project*.

Four quantitative measures were also derived from the social network analysis: network density, in-degree centrality, betweenness centrality, and homophily. All social network analysis were conducted using UCINET (version 6) [24].

### Network density

Network density is defined as the number of connections in a network reported as a proportion of the total links possible based on the number of actors in the network. It ranges from 0 to 1 where 0 connotes no relationships between the organizations within a network and 1 refers to every organization having a link with all other organizations. High network density is linked to the spread of new information as well as better understanding between actors, thus theoretically leading to more effective solutions [19, 25], although some argue that this relationship is likely curvilinear, whereby too dense of a network can also hinder an organization’s ability to access information from outside of the network [16, 19].

### Network centrality

Network centrality is used to identify the most central and influential organizations within a network. In this study, we measured both in-degree centrality and betweenness centrality. In-degree centrality calculates the number of times an organization is nominated by other organizations in the network and is used to identify opinion leaders [16]. In this study, an organization is “nominated” when another organization reported having worked with that organization on MCH and/or WASH.

Betweenness centrality counts the frequency that an organization lies on the shortest path between two other organizations, divided by the maximum possible value [16]. An organization with high betweenness centrality can serve as a gatekeeper as it has the ability to control the flow of information as well as resources between other organizations in the network.

### Homophily

To better understand interactions between organizations working on MCH and WASH, the measure homophily was explored. In this case, homophily refers to the tendency that organizations from MCH, for example, interact more frequently with each other than with organizations from WASH and vice versa. Homophily ranges from -1 to 1 where -1 connotes complete homophily and 1 refers to complete heterophily [16].

For the qualitative component, recorded in-depth interviews and notes were converted to textual form through transcription. Interviews conducted in Hindi were transcribed and then translated into English. Subsequently, thematic analysis was used to identify common themes across informants.

### Ethical approval

This study received ethical approval from the Johns Hopkins School of Public Health’s Institutional Review Board in Maryland, USA and the Indian Institute of Health Management Research’s Institutional Review Board in Jaipur, India. Informed verbal consent was obtained from the key informants before conducting the interviews. The interviews only proceeded after consent was obtained. The use of oral consent was approved because the study was deemed minimal risk.

## Results

A total of 28 organizations were identified and 31 key informants from 24 organizations agreed to participate in the study (response rate: 85.7%). These informants included representatives from governmental, non-governmental, multilateral, and international organizations. Five were solely involved in WASH activities, 14 in MCH, and 5 were engaged in both sectors. The majority of the organizations have worked in the area for less than 5 years (N = 13) or more than 15 years (N = 10). A summary of the organizations’ characteristics is illustrated in Table 1.

**Table 1:** Characteristics of organizations.

	Frequency	Percent (%)
Sector		
• Health	14	58%
• WASH	5	21%
• Both	5	21%
Organizational Affiliation		
• Public	9	38%
• Private (i.e. NGOs, Foundations)	9	38%
• International/Multilateral	6	25%
Years working Kachhauna, Hardoi		
• Less than 5 years	13	54%
• 5 to 15 years	1	4%
• More than 15 years	10	42%

### Network structure

**Fig 1:**
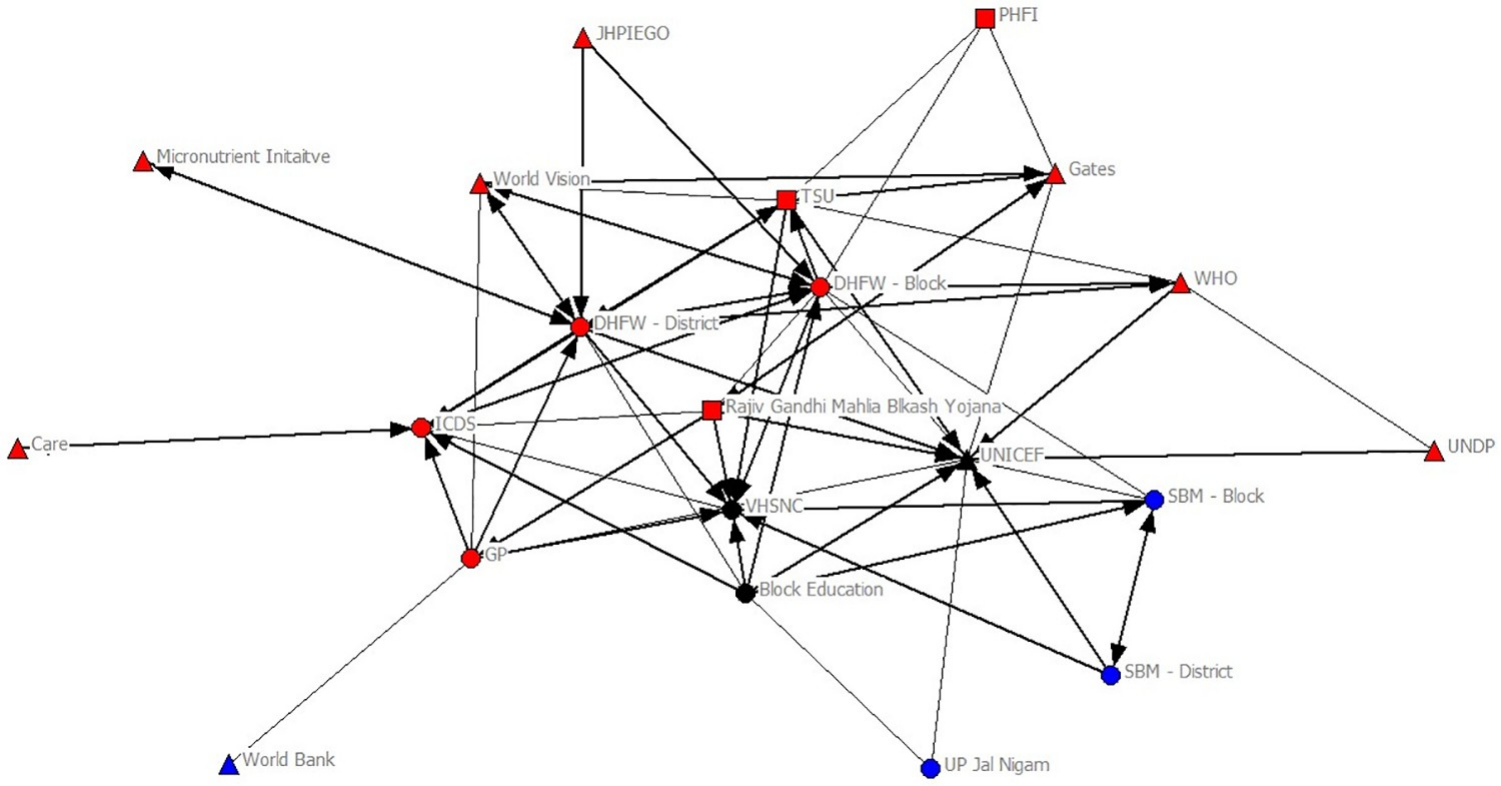
Network before HCL Samuday Project. Red: Health; Blue: WASH; Black: Health & WASH; Triangle: International/Multinational; Circle: Public; Square: Private.

**Fig 2:**
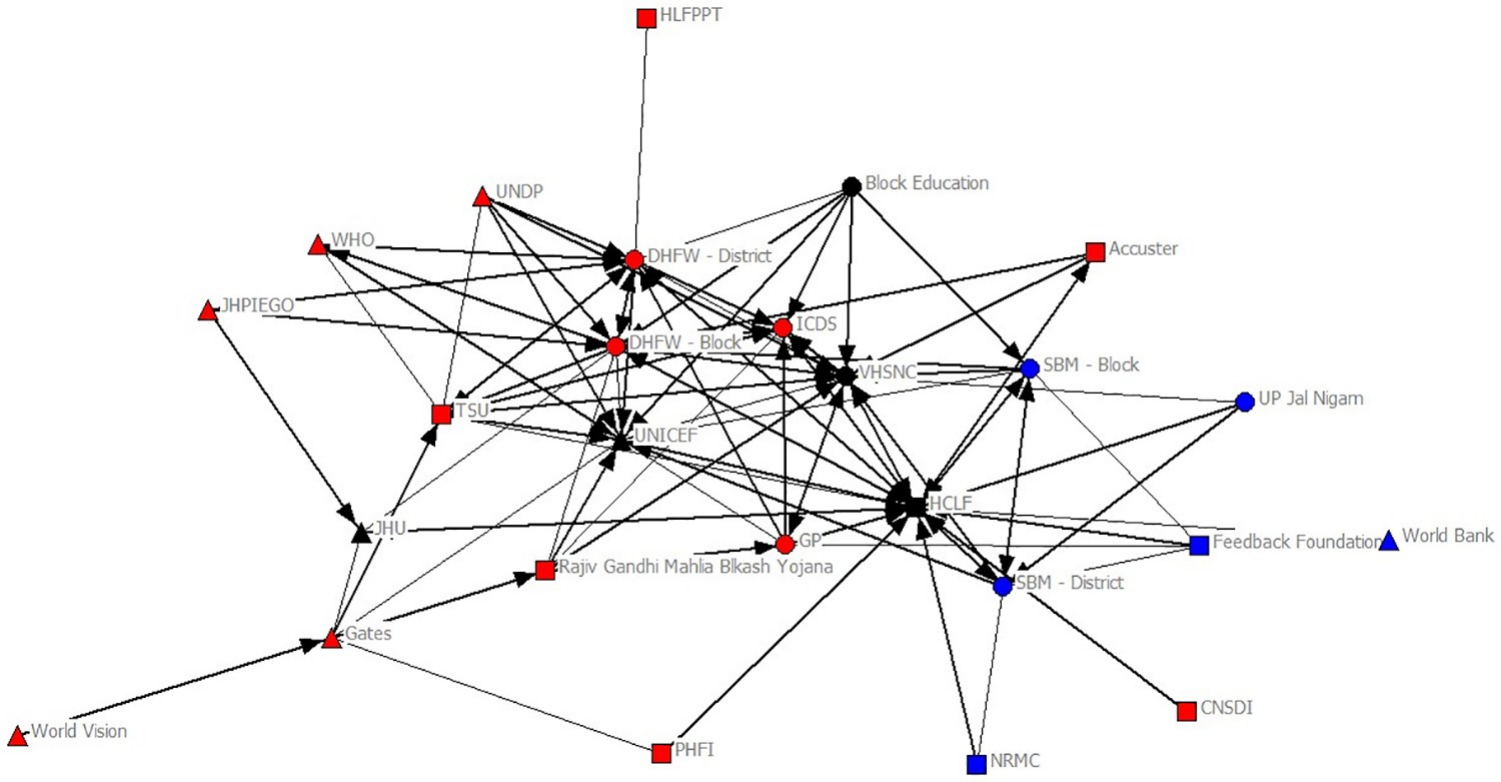
Network after HCL Samuday Project. Red: Health; Blue: WASH; Black: Health & WASH; Triangle: International/Multinational; Circle: Public; Square: Private.

The sociograms in Figs 1 and 2 provide a visual illustration of the changes in the network prior to the implementation of *HCL Samuday Project* and after the inception of the project. The number of organizations increased from 21 (Public: 9; Private: 3; International: 9) to 26 (Public: 9; Private: 9; International: 8) after the implementation; specifically, 6 new organizations (Private: 5; International 1) were introduced into the network. Network density, on the other hand decreased from 0.293 to 0.278, connoting that the number of connections in the network reported as a fraction of the total links possible reduced slightly (Table 2).

**Table 2:** Network characteristics before and after HCL Samuday Project.

	Before HCL Samuday Project	After HCL Samuday Project
Network Density	0.293	0.278
Network Centrality	UNICEF: 12	HCLF: 15
	VHSNC: 9	VHSNC: 13
	Dep. of Health–District: 8	UNICEF: 13
	Dep. Of Health–Block: 8	Dep. Of Health–Block: 9
Homophily	-0.915	-0.902

### Network centrality

Prior to *HCL Samuday Project*, the organizations with the highest in-degree centrality were UNICEF, the Village Health, Sanitation, and Nutrition Committee (VHSNC), Department of Health–District, and Department of Health–Block; these organizations received 12, 9, 8, and 8 nominations respectively. After *Samuday*, the organizations with the highest in-degree centrality were HCLF, VHSNC, UNICEF, and Department of Health–Block; these organizations received 15, 13, 13, and 9 nominations respectively. The in-degree centrality for VHSNC, specifically, increased from 9 to 13 (Table 2).

**Fig 3:**
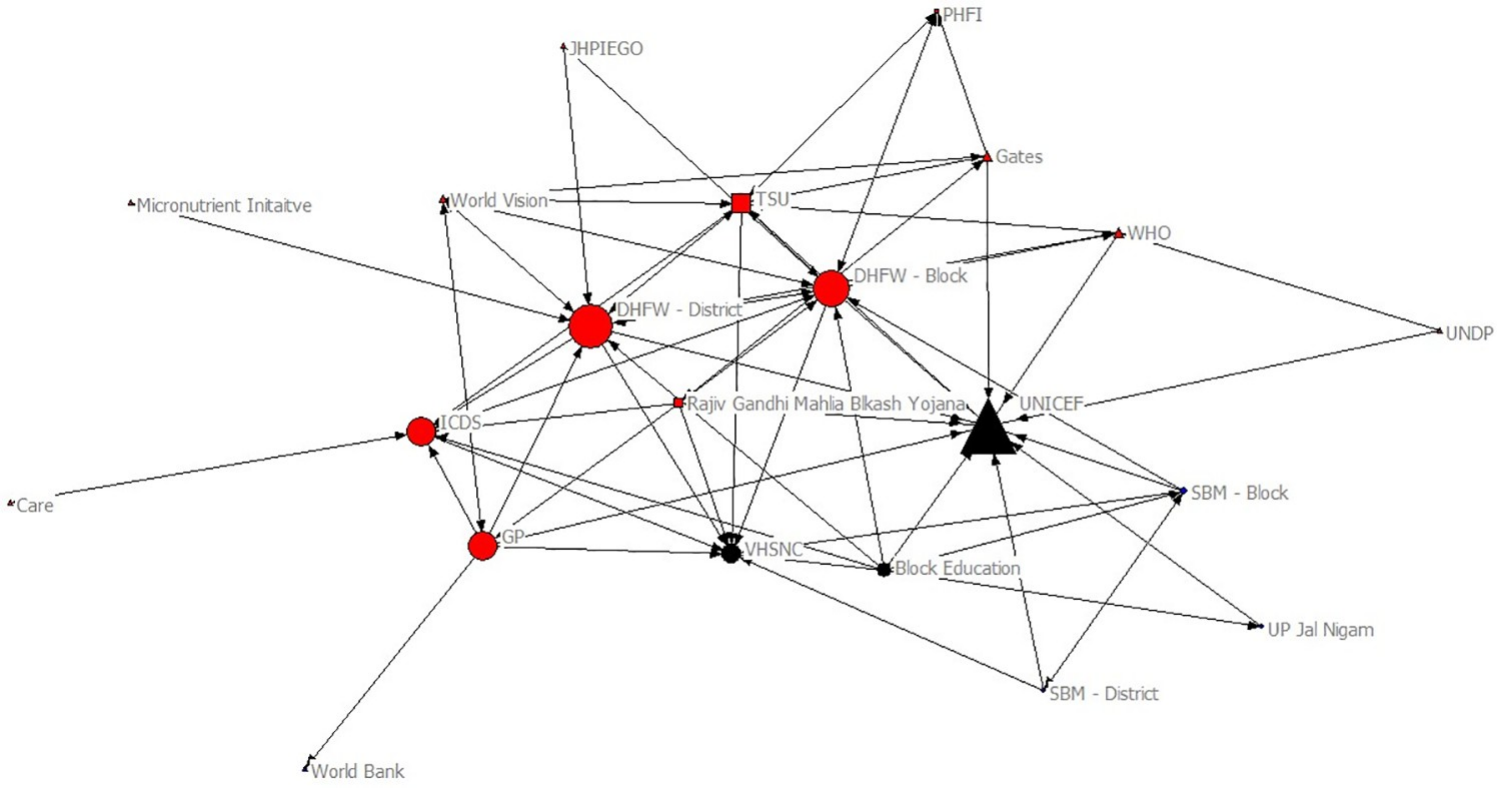
Betweeness Centrality before HCL Samuday Project. Triangle: International/Multinational; Circle: Public; Square: Private.

**Fig 4:**
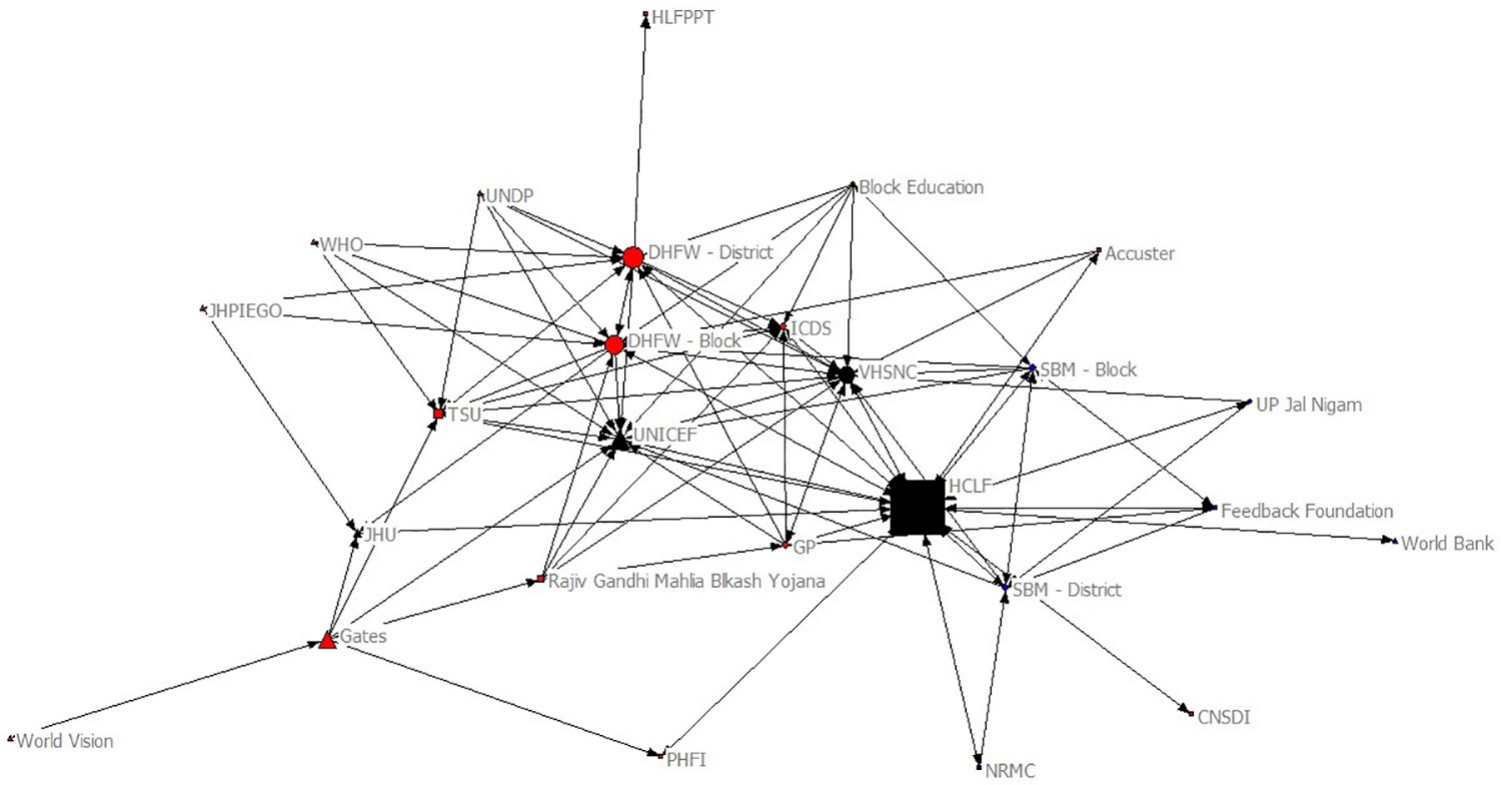
Betweeness Centrality after HCL Samuday Project. Triangle: International/Multinational; Circle: Public; Square: Private.

The sociograms in Figs 3 and 4 provide a visual illustration of the organizations’ betweenness centrality scores before and after the implementation of the project; the larger the organization is portrayed in the figure, the higher the betweenness centrality score. Accordingly, UNICEF, Department of Health–District Level, and Department of Health–Block Level had the highest betweenness centrality scores before *HCL Samuday Project* and HCLF, Department of Health–District Level, and Department of Health–Block Level had the highest scores after *Samuday*.

### Homophily

When examining the interactions between organizations working in the MCH (N = 14) and WASH (N = 5) only, this study found that the homophily score was -0.915 before the project and -0.902 after the implementation of the project (Table 2).

Consistent with our findings on homophily, while some informants lauded *Samuday* for providing a better platform to interact with other organizations, many highlighted the need for more interactions across sectors as well as between organizations in the form of information sharing and regular meetings, highlighting that if inter-organizational interactions increased, they would be able to work more effectively, enhance coordination, find solutions to problems more readily, share project updates and avoid duplication:

“*The frequency of the meetings among the departments or organizations should be increased. Secondly, the HCL should share its finding or the shortcomings it notices anywhere during the work. Through such sharing we will stay updated and at the same time they will also get good support from our side*”

Government Official

“*I think it is a good suggestion to organize a meeting of all partners. Solutions of many issues, I think, could be sought just in that meeting … At present, we don’t have any platform like this, but if it is done in future, it would be beneficial*”

Private Organization

Two informants also pointed to the need to diversify the network by engaging with civil society organizations (CSO). Currently, CSOs working in the same district are often not aware of each other or each other’s work. Moreover, it is challenging for CSOs to access governmental organizations:

“Government doors aren’t so open for CSO. CSO can bring new perspective but government so tied to its own procedures”

*International*/*Multilateral Organization*

## Discussion

This study used SNA to provide a visual snapshot supplemented by contextualized narrative about the relationships between the organizations working on MCH and WASH in a rural community in India. As few studies have used SNA in monitoring stakeholder relationships, intervening, improving and facilitating project implementation involving intersectoral partnerships in LMICs, our study also helped demonstrate the utility and feasibility of using SNA to this effect. We showed that SNA can be used as a multi-purpose strategic planning and management tool for projects like *Samuday* as it 1) helps map the context, including key organizational actors in a defined network b) provides a framework for planning collaboration, c) creates a visual representation of the key interactions, and d) allows for the monitoring of the status of those relationships on a regular basis.

Findings from this study revealed that the network of organizations working on MCH and WASH grew since the implementation of *HCL Samuday Project*; five new organizations are now involved in one or both sectors in Kachhauna. As an inverse relationship exists between size and density (Valente, 2010), the introduction of new actors can help explain the overall density decrease (from 0.293 to 0.278).

HCLF has also rapidly achieved centrality and can potentially serve as a gatekeeper of information between different sectors and organizations. Homophily scores revealed that organizations involved in MCH and WASH primarily collaborated within their own thematic sector (e.g., health, water) at both time points. This was highlighted by key informants during the interviews, with many expressing a high level of interest in more interorganizational interactions and engagement.

This finding is consistent with existing studies that had explored inter-organizational collaboration in India, both between different organizational types and between different thematic sectors. Srivastava et al (2016), for example, revealed that there is only a moderate level of linkage between the public and non-government organizations in healthcare in Uttar Pradesh [26]. Specifically, data sharing as well as NGO participation in health planning activities were limited despite the importance of NGOs in service provision to vulnerable Indian communities. Likewise, Kim et al (2017), revealed that while collaboration between health and nutrition was present at the state level, it was lacking at the block level in Odisha, India. This, they explained, was as a result of inadequate resources, heavy workload, poor communication and lack of direction as well as guidelines [27].

From a theoretical perspective, the high network centrality of the HCLF at the second time point is consistent with the role of a centralized network broker or “lead organization”, which serves a governance function for the network and/or facilitates interactions between network members to achieve collective goals [28]. This type of centralized “weaver” role is common in the early stages of a network development process to facilitate information exchange, resource sharing and trust-building until members agree on how to operationalize the network [29–30].

It is important to note that there are some challenges, practical considerations, and limitations associated with the use of SNA in this context. First, in our study, *snowball sampling* was used since a comprehensive list of organizations working on MCH and/or WASH in Kachhauna did not exist. This sampling approach may be time-consuming, and organizations not directly involved in the project can be, at times, difficult to access. Moreover, there may be bias as the sample is dependent on the choice of the initial informants interviewed. These individuals may have nominated organizations with larger networks and missed those with smaller networks. However, given that Kachhauna is a relatively small area geographically, it is likely that we were able to identify and capture the majority of the organizations in the network.

Despite the fact that the response rate was high (85.7%) not all organizations agreed to participate. As such, there were missing data from some of the organizations. Missingness can be particularly concerning for SNA as it connotes that inferences are being made from a partial network. Fortunately, there are some helpful rule of thumbs available for practitioners and evaluators interested in this approach, including the use of more robust centrality measures when response rates are 70% or less and employing simple imputation procedures to address missingness [31–33].

Given that we sought to characterize collaboration taking place *in practice* rather than *on paper*, so to speak, we did not differentiate between formal and informal collaborative interactions, though we acknowledge that such differentiation may be useful for tracking the institutionalization of intersectoral collaboration over time. Moreover, we also excluded entities with only an auxiliary or indirection function, such as supplying products or commodities to one of the collaborating organizations.

Finally, there may be recall bias as informants were asked to recall their relationships with other organizations before *HCL Samuday Project* was launched. In an ideal scenario, SNA would first be undertaken at baseline, prior to the start of the project, and then carried out periodically throughout the implementation of the project. This type of longitudinal network analysis will help address both the inherent dynamic nature of networks and prevent recall bias associated with retrospective inquires. It will also help project implementers identify the elements of the network structure that is associated with intervention effectiveness.

## Conclusions

This is one of the few studies that utilize an organizational-level social network analysis to characterize intersectoral collaboration in the context of a low-and middle-income country. While there are limitations and practical considerations associated with this approach, this study showed that SNA is an innovative tool that can provide valuable insight into the interactions between organizations working in different sectors, assist program implementers strategically plan, promote and monitor intersectoral collaboration for health, and facilitate in the identification of strategies to strengthen collaboration and increase heterophily.

Future work should track the evolution of networks prospectively to identify the elements most strongly associated with intervention effectiveness. Moreover, there is also a need to further test the assumption that increased interactions among different sectors would lead to better social and economic development in rural areas.

## Supporting information

S1 File Social network data. (XLSX)
